# Association between periodontal inflamed surface area and estimated glomerular filtration rate among pre-dialysis chronic kidney disease patients

**DOI:** 10.11604/pamj.2025.51.69.43890

**Published:** 2025-07-08

**Authors:** Olusoji Ayodele Onabanjo, Solomon Olusegun Nwhator, Fatiu Abiola Arogundade, Opeyemi Matthew Adewole, Tolulope Ogundiran, Babatope Andrew Ogunleye

**Affiliations:** 1 Obafemi Awolowo University Teaching Hospitals Complex, Ile Ife, Osun State, Nigeria,; 2 Obafemi Awolowo University Ile Ife, Osun State, Nigeria

**Keywords:** Periodontitis, kidney disease, kidney function, inflammation

## Abstract

**Introduction:**

periodontitis increases systemic inflammatory burden and the effect of this could be worsening renal function of chronic kidney disease patients (CKD). The purpose of this study was to evaluate the relationship between renal function and periodontal inflammation among CKD population attending a tertiary institution in Nigeria.

**Methods:**

a total of 120 pre-dialysis CKD patients were enrolled in this study and their serum creatinine level was measured for the estimation of glomerular filtration rate (eGFR). The burden of periodontal inflammation was evaluated using periodontal inflamed surface area (PISA). Simple and Multiple linear regression analysis was done to explain the relationship between renal function and periodontal inflammation.

**Results:**

a total number of 120 participants, made up of 68 (56.7%) males and 52 (43.3%) females diagnosed of chronic kidney disease (CKD). On simple and multiple linear regression analysis, PISA was significantly associated with eGFR (β = -0.017, 95% CI -0.026 to -0.009; P < 0.001). Pearson correlation analysis also suggested a moderate correlation between PISA and eGFR (r=-0.5, p<0.001).

**Conclusion:**

this study suggests that renal function and periodontal inflammation might be related, increasing the severity of periodontitis, which could possibly lead to decreased renal function.

## Introduction

Chronic kidney disease (CKD) is defined as abnormalities of kidney structure or function for 3 months or more [1,2]. Chronic kidney disease has become a public health problem, evident by its global prevalence and increasing morbidity and mortality, especially in developing countries like Nigeria. It is estimated that by 2030, more than 2 million people in the United States of America will be on dialysis or need a kidney transplant. The severity of CKD is grouped into 5 stages based on Glomerular Filtration Rates (GFR) [3]. The grouping criteria were developed by the National Kidney Foundation as part of its Kidney Disease Outcomes Quality Initiative (NKF KDOQI) [4]. Glomerular filtration rate (GFR) is generally accepted as the best overall measure of kidney function in health and disease [5,6].

Periodontitis is an infection-induced inflammation of the supporting tissues of the teeth, resulting in progressive bone and attachment loss [7]. The clinical hallmark of periodontitis is a pathologic pocket and or gingival recession, and eventual tooth loss if left unattended. Inflammation plays a vital role in the mechanism and progression of CKD [8]. Inflammation has been linked to several causes like dietary and lifestyle factors, oxidative stress, dialysis-related factors, intestinal dysbiosis, immune dysfunction, as well as periodontal disease [8]. Periodontal diseases, as one of these sources of inflammation, have often been underestimated. One of the recent Nigerian studies revealed that 91% of CKD patients had periodontal diseases [9]. Periodontal inflammation increases the burden of systemic inflammation in CKD by releasing cytokines and this in turn leads to worsening renal function in these patients [10]. Inflammation is also a major contributory factor to the two leading causes of death (cardiovascular diseases and infections) among CKD patients [8,11]. Therefore, the purpose of this study was to evaluate the relationship between renal function and periodontal inflammation among CKD population attending a tertiary institution in Nigeria.

## Methods

**Study design and setting:** this was a cross-sectional study that evaluated the relationship between renal function and periodontal inflammation among CKD population attending a tertiary institution in Nigeria.

**Study population:** the participants enrolled in this study include 120 chronic kidney disease patients who were undergoing conservative management. All the participants provided written and informed consent. Patients who have diabetes mellitus or diabetic nephropathy, those with any form of immunosuppression, or who have had periodontal treatment in the last six months were excluded from the study.

**Data collection:** periodontal examination was carried out, and probing pocket depth (PPD), clinical attachment level (CAL), recession and bleeding on probing (BOP) measurements were recorded. These were used to calculate periodontal inflamed surface area (PISA).

**Laboratory analysis:** blood samples were collected for serum creatinine for the estimation of GFR. Blood sample collected was centrifuged at 3000 rev/sec for 10 minutes and the serum obtained was stored at -80°C for storage. Serum creatinine was analysed according to Randox Laboratories Ltd test kit instructions. GFR was estimated from serum creatinine using the Cockcroft-Gault (CG) equation, Ccr = [(140 -age) × weight/] (72 × Scr) × 0.85 (if the subject is female), where Ccr is expressed in milliliters per minute, age in years, weight in kilograms, and serum creatinine (Scr) in milligrams per decilitre.

**Statistical analysis:** data collected were analysed using IBM SPSS Statistics 23.0 and STATA 17.0. Descriptive analysis was carried out for socio-demographic variables such as age, sex, and educational attainment. Association between PISA and eGFR was analysed using the Pearson's Correlation test, while comparison between periodontal status and renal function was analysed with the independent t-test. To explore the association between periodontal inflammation and kidney function, a simple linear regression was first conducted with estimated glomerular filtration rate (eGFR) as the dependent variable and periodontal inflamed surface area (PISA) as the predictor. This was followed by a multiple linear regression analysis to adjust for potential confounders, including age, sex, socio-economic status and hypertension. A p-value of <0.05 was considered statistically significant. Interpretation of results focused on the magnitude and direction of the adjusted beta coefficients, their 95% confidence intervals, and P-values, to determine the strength and significance of associations between PISA and eGFR while controlling for other factors.

**Ethical considerations:** the Ethics and Research Committee (ERC) of the Obafemi Awolowo University Teaching Hospitals Complex, Ile Ife, Osun State, Nigeria, approved all study protocols, which were per the principles of the Declaration of Helsinki. Written and verbal informed consent were obtained from all participants before their enrollment in the research.

## Results

**General characteristics of study population:** a total of 120 participants, made up of 68 (56.7%) males and 52 (43.3%) females diagnosed of CKD, who met the inclusion criteria, were recruited into the study. The mean age of the participants was 45.7±16.63 years.

***Gender comparison of the mean values of oral hygiene, periodontal status, and renal function:*** the males had significantly higher values of OHIS, PPD, CAL, PISA compared to the females (p<0.01) (Table 1). However, the males had significantly lower values of eGFR than the females at baseline (p<0.01).

**Table 1:** gender comparison of the mean values of oral hygiene, periodontal status, and renal function at baseline

Indices	Gender	N	Mean	SD	T	P
**Baseline OHI-S**	Male	68	2.89	1.11	3.39	<0.01
	Female	52	2.22	1.05	-	
**Baseline PPD Mm**	Male	68	4.21	1.77	3.61	<0.01
	Female	52	3.10	1.59	-	
**Baseline CAL Mm**	Male	68	4.75	2.25	3.51	<0.01
	Female	52	3.37	2.05	-	
**Baseline PISA mm^2^**	Male	68	476.82	414.90	2.92	<0.01
	Female	52	262.37	385.21	-	
**Baseline eGFR ml/min**	Male	68	46.16	19.92	-3.49	<0.01
	Female	52	58.06	17.32	-	
T = Independent T test, SD = Standard Deviation						

***Comparison of the mean values of PISA and eGFR between Group 1 and Group 2:*** those with periodontitis had notably higher mean PISA score than those without periodontitis (p<0.01) (Table 2). Also, participants with good oral hygiene (those without periodontitis) had significantly higher mean eGFR than those with moderate to severe periodontitis (p< 0.01) (Table 2).

**Table 2:** comparison of the mean values of PISA and eGFR between Group 1 and Group 2 at baseline

Markers	Group	N	Mean	SD	T	P	95% CI	
							Lower	Upper
**PISA (mm^2^)**	Group 1	60	711.60	357.58	14.17	<0.01	563.84	746.98
	Group 2	60	56.19	20.89				
**eGFR (ml/min)**	Group 1	60	40.55	17.16	-7.14	<0.01	-27.49	-15.55
	Group 2	60	62.08	15.84				
Independent t-test, SD= Standard Deviation								

***Pearson's correlation analysis- relationship between renal function and periodontitis:*** Table 3 shows a moderate correlation between renal function and periodontitis as assessed by PISA and eGFR, respectively (r= -0.5, p<0.001) at baseline.

**Table 3:** relationship between periodontitis (using PISA) and renal function (using eGFR) in pre-dialysis CKD patients

Test	Variable	PISA (mm^2^)	eGFR (ml/min)
**PISA**	R	1	-0.528**
	P		<0.001
	N	120	120
**eGFR**	R	-0.528**	1
	P	<0.001	
	N	120	120
Pearson Correlation Analysis **Significant correlation			

**Simple linear regression analysis- Unadjusted relationship between eGFR and PISA:** PISA Coefficient is -0.025. (Table 4). This means that for every 1 mm^2^ increase in PISA, the eGFR decreases by approximately 0.025 mL/min/1.73m^2^, without adjustment for any other variables. There is a significant inverse association between PISA and eGFR in the unadjusted model. As periodontal inflammation (PISA) increases, renal function (eGFR) tends to decrease. Specifically, each unit increase in PISA is associated with a 0.025 unit decline in eGFR (p < 0.001).

**Table 4:** factors that might influence eGFR

	eGFR			
**Factors**	Unadjusted β Coefficient (95% CI)	P-value	Adjusted β Coefficient (95% CI)	P-value
**PISA**	-0.025(-0.0324 to -0.0177)	<0.001	-.0173(-0.026 to -0.008)	<0.001
**Age**	-0.466(-.664 to -0.268)	<0.001	-0.2681(-.592 to 0.056)	
**Sex**				
Female	11.90(5.028 to 18.776)	<0.001	7.8962(1.701 to 14.091)	0.013
**Socio-economic class**				
III	7.25(-3.854 to 18.374)	0.198	5.4743(-4.383 to 15.332)	0.274
IV	5.94(-5.457 to 17.340	0.304	9.4973(-0.81566 to 19.8)	0.071
V	21.13(8.497 to 33.772)	0.001	12.701(.16 to 25.24)	0.047
**Hypertension**				
Yes	-6.614(-1.739 to 49.715)	0.068	4.2405(-3.696 to 12.177)	0.292

**Multiple Linear Regression analysis- Factors that might influence eGFR:** a multiple linear regression model was constructed to assess the independent association between PISA and eGFR while adjusting for potential confounders. This included age, sex, socio-economic status and the presence of comorbidities like hypertension and categorical variables were included using appropriate factor variable notation. PISA was significantly associated with eGFR (β = -0.017, 95% CI -0.026 to -0.009; p < 0.001) (Table 4). This suggests that for each unit increase in PISA, eGFR decreases by approximately 0.017 units, after adjusting for other variables. Age showed a non-significant negative association with eGFR (β = -0.27, 95% CI -0.593 to 0.056; p = 0.104) (Table 4). Although not statistically significant, the trend indicates that older age may be associated with reduced eGFR. Sex (Female) was significantly associated with higher eGFR compared to males (β = 7.90, 95% CI 1.70 to 14.09; p = 0.013) (Table 4). This suggests that females had, on average, a 7.9-unit higher eGFR than males, adjusting for other factors. Compared to the reference group (SES II), SES V showed a significant positive association with eGFR (β = 12.70, 95% CI 0.16 to 25.25; p = 0.047) (Table 4), indicating that individuals in SES V had significantly higher eGFR. Age and hypertension were not statistically significant in this model but showed trends in the expected direction.

## Discussion

The objective of this study was to evaluate the relationship between renal function and periodontal inflammation among the CKD population attending a tertiary institution in Nigeria. This study determined whether PISA as a predictor of periodontal inflammation, correlates with estimated glomerular filtration rate (a marker of renal function). In this study, the baseline mean PISA value was more than tenfold higher in the CKD group with periodontitis than in those without periodontitis. PISA value was also almost two times higher in male participants compared to females. CKD participants without periodontitis had higher values of eGFR compared to those who had periodontitis. There was also a moderate correlation between PISA and eGFR with Pearson's Correlation Analysis. Simple linear regression analysis suggested that there was a decline in the eGFR with every rise in PISA value and the multiple linear regression model suggested that for each unit increase in PISA, eGFR decreases by approximately 0.017 units, after adjusting for other variables. The model also showed that age had a non-significant negative association with eGFR. However, the trend in the model indicated that older age may be associated with reduced eGFR. Female participants have higher eGFR compared to males. This was on average about 7.9 units higher than males after adjusting for other factors. Meanwhile, socio-economic status had a significant positive association with eGFR, especially with SES V, who are mainly students.

Increased PISA values in the CKD group with periodontitis are a reflection of the possible burden of periodontal inflammation on systemic inflammation in these patients, which in turn has an effect on their renal function. This is consistent with other studies, which also reported significantly higher values of PISA in association with increasing periodontal disease severity [12-15]. The male participants have poor oral hygiene compared to the female and that could explain the twofold increase in PISA values for males compared to females. Increased burden of inflammation from periodontitis on systemic inflammation could possibly explain lower eGFR values found in CKD participants with periodontitis as opposed to those without periodontitis. So, there seems to be a correlation between PISA and eGFR. Once periodontal inflammation increases, patients are likely to suffer a decline in their renal function.

In exploring the association between PISA and eGFR, potential confounders such as age, sex, socio-economic status and comorbidity (hypertension) were adjusted for in the multivariate analysis and we found that for every unit rise in PISA, there would be a corresponding decline in eGFR by approximately 0.017 unit. These findings in our study are in agreement with other studies [16-19]. Schütz *et al*. [16] reported that individuals with severe periodontitis have a higher risk of being in stages 4 and 5 of CKD after adjusting for major confounders. Worst periodontal condition was found to be associated with higher severity of CKD in pre-dialysis patients. Iwasaki *et al*. [17] found that in some Japanese elders, those with advanced periodontal inflammation had an increased risk of renal dysfunction over 2 years of follow-up. Sharma *et al*. [19] reported that individuals with stages 3-5 pre-dialysis CKD were more likely to have severe periodontitis. All these findings are consistent with the results of our study.

This association between periodontitis and chronic kidney disease could possibly be explained by a persistent immune response following periodontal infection, which now leads to low-grade systemic inflammation. This low-grade systemic inflammation causes renal dysfunction [16]. Periodontal pathogens also spread via the bloodstream, resulting in systemic inflammation, which eventually leads to decreased renal function [16]. Determining the association between PISA and eGFR using PISA as an index of periodontitis is a better approach at quantifying the burden of inflammation and is a major strength of this study. However, the limitation is that the strength of this association is still a subject of debate because of many factors shared by both periodontitis and chronic kidney disease, which include age, smoking, diabetes mellitus, and cardiovascular diseases. Also, severe CKD patients, because of the uraemic dysregulation of the immune system, become susceptible to infections such as periodontitis [18].

## Conclusion

This study suggested a possible relationship between renal function and periodontitis. An increase in the severity of periodontitis leads to decreased renal function. Reducing the burden of local inflammation on systemic inflammation through periodontal therapy may prove efficient in improving renal function.

### What is known about this topic

- That periodontitis possibly has an impact on renal function;
- This impact has been assessed using periodontal status parameters such as probing pocket depth (PPD) and clinical attachment loss (CAL);
- These PPD and CAL are linear measurements that do not quantify the inflammatory burden of periodontitis on renal function.

### What this study adds

- Determining the relationship between renal function and periodontitis using PISA as an index of periodontitis is a better approach at quantifying the burden of inflammation;
- PISA quantifies the burden of periodontal inflammation added to the overall systemic inflammation;
- This additional burden of local inflammation from periodontitis, which PISA assesses more accurately, gives a better picture of the impact of periodontitis on renal function.
